# Greater numbers of nucleotide substitutions are introduced into the genomic RNA of bovine viral diarrhea virus during acute infections of pregnant cattle than of non-pregnant cattle

**DOI:** 10.1186/1743-422X-9-150

**Published:** 2012-08-06

**Authors:** John D Neill, Benjamin W Newcomer, Shonda D Marley, Julia F Ridpath, M Daniel Givens

**Affiliations:** 1 Ruminant Diseases and Immunology Research Unit, National Animal Disease Center, USDA, ARS, 1920 Dayton Ave, Ames Iowa 50010, USA; 2 Department of Pathobiology, College of Veterinary Medicine, Auburn University, Auburn, AL 36849, USA

**Keywords:** Persistent infection, Virus genetic diversity, Nucleotide substitutions

## Abstract

**Background:**

Bovine viral diarrhea virus (BVDV) strains circulating in livestock herds show significant sequence variation. Conventional wisdom states that most sequence variation arises during acute infections in response to immune or other environmental pressures. A recent study showed that more nucleotide changes were introduced into the BVDV genomic RNA during the establishment of a single fetal persistent infection than following a series of acute infections of naïve cattle. However, it was not known if nucleotide changes were introduce when the virus crossed the placenta and infected the fetus or during the acute infection of the dam.

**Methods:**

The sequence of the open reading frame (ORF) from viruses isolated from four acutely infected pregnant heifers following exposure to persistently infected (PI) calves was compared to the sequences of the virus from the progenitor PI calf and the virus from the resulting progeny PI calf to determine when genetic change was introduced. This was compared to genetic change found in viruses isolated from a pregnant PI cow and its PI calf, and in three viruses isolated from acutely infected, non-pregnant cattle exposed to PI calves.

**Results:**

Most genetic changes previously identified between the progenitor and progeny PI viruses were in place in the acute phase viruses isolated from the dams six days post-exposure to the progenitor PI calf. Additionally, each progeny PI virus had two to three unique nucleotide substitutions that were introduced in crossing the placenta and infection of the fetus. The nucleotide sequence of two acute phase viruses isolated from steers exposed to PI calves revealed that six and seven nucleotide changes were introduced during the acute infection. The sequence of the BVDV-2 virus isolated from an acute infection of a PI calf (BVDV-1a) co-housed with a BVDV-2 PI calf had ten nucleotides that were different from the progenitor PI virus. Finally, twenty nucleotide changes were identified in the PI virus of a calf born to a PI dam.

**Conclusions:**

These results demonstrate that nucleotide changes are introduced into the BVDV infecting pregnant cattle at rates of 2.3 to 8 fold higher then during the acute infection of non-pregnant animals.

## Background

Bovine viral diarrhea virus (BVDV) is a member of the Pestivirus genus of the family *Flaviviridae*. BVDV strains isolated from domestic livestock herds show considerable variability in nucleotide and amino acid sequences. This variation led to the division of BVDV into two species, BVDV1 and BVDV2, and further divided type 1 viruses into more than 12 subgenotypes and type 2 viruses into 2 subgenotypes [1-3]. The source of this variation is believed to be due to an error-prone, non-proofreading RNA-dependent RNA polymerase. Because of this, BVDV exists as a quasispecies, each viral genome possessing a small number of nucleotide and amino acid differences from the viral population consensus sequence [4,5]. This sequence diversity allows a virus population to adapt to environmental stresses, such as immune pressure from an adaptive immune response or to quickly develop drug resistance [6-8]. A virus with amino acid substitutions that confer resistance or a growth advantage will quickly become the dominant virus in the population.

An unique feature of noncytopathic BVDV strains are their ability to establish lifelong, immunotolerant infections when a pregnant dam is infected in the first trimester of pregnancy and before maturation of the fetal immune system. Acute infection of the dam is followed by transmission of the virus to the fetus at roughly 14 days post infection [9]. The dam clears the infection and produces an immune response against the virus while its calf is born persistently infected (PI) and immunotolerant of the infecting virus. The PI animal sheds the virus for the remainder of its life. It was assumed that the persistent virus possessed very few nucleotide differences from the virus infecting the dam [10] but sequence variation was shown to occur over time in the PI animal [4]. Additionally, a study examining nucleotide and amino acid changes that were introduced following establishment of persistent infections using a biologically cloned virus inoculum was conducted [5]. This study resulted in production of two PI calves that were sampled at seven and 18 months of age. This analysis showed that small numbers of nucleotide substitutions were present in the E2 protein, with some resulting in amino acid changes. Interestingly, more variants were identified with increasing age of the animals. These studies demonstrated that BVDV exists as a quasispecies in the PI animal and that genetic change was introduced during the infection of the fetus and that additional changes occurred with time.

This study was done to more clearly define when and to what degree genetic change was introduced into the BVDV genomic RNA. There were a greater number of nucleotide substitutions in viruses from PI calves whose dam had been exposed to a PI calf during the first trimester of pregnancy then during a BVDV outbreak caused by a single strain of BVDV followed over more than a year [11]. To answer when the greatest amount of genetic change was introduced, the sequences of viruses isolated from progenitor PI calves, pregnant dams and their progeny PI calves were compared to the number of change in viruses isolated from acute infections in non-pregnant, BVDV naïve cattle exposed to PI calves.

## Materials and methods

### Cells and virus propagation

Madin-Darby bovine kidney (MDBK) cells were maintained in Eagle’s MEM supplemented with 1.4 mM sodium bicarbonate and 10% BVDV virus and BVDV antibody free fetal calf serum at 37°C in a 5% CO_2_ atmosphere. BVDV strains from acutely and persistently infected animals were isolated from serum or buffy coat by a single passage on MDBK cells to limit possible genetic changes that may be introduced by in vitro passage.

### Viruses

**Figure 1:**
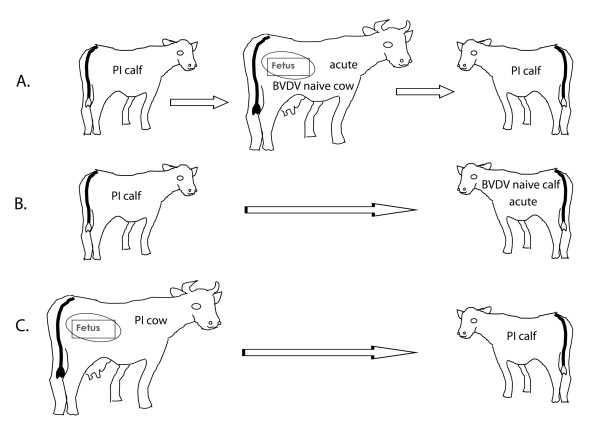
**Experimental overview and source of viruses. A**. BVDV-naïve, pregnant dams in the first trimester of pregnancy were exposed to PI calves. Viruses were isolated from the progenitor and progeny PI calves and from the dams six days post-exposure. **B**. BVDV naïve cattle were exposed to PI calves. Viruses were isolated from the PI calves and from the acutely infected cattle at 13 days post-exposure. **C**. Viruses were isolated from a PI dam and its PI calf.

The viruses used in this study and their relationships are shown in Table1 and Figure1. The ORF sequences of the progenitor PI viruses 180, 446, 526 and progeny PI viruses 8824, 8827, 8831, 8833 and 8844 were reported previously [11]. All viruses were named with the animal number from which the virus was isolated. This experiment (Figure1a) was conducted by exposing pregnant heifers at approximately 70 days of pregnancy to PI calves (one calf each with a BVDV1a, 1b and 2) and isolating virus from the heifers at 6 days post-exposure and from calves at birth [12]. The PI virus 180 (1a) gave rise to viruses 6010 and 8844; PI virus 526 (1b) gave rise to 6151 and 8824; and PI virus 446 (2) gave rise to viruses 6136 and 8827 and 6115 and 8831. An additional set of viruses from a pregnant animal (Figure1c) was obtained by isolation of the virus from a pregnant PI cow and from her heifer calf at birth (viruses Powder and PJ).

**Table 1:** Viruses used in this study

**Progenitor PI Viruses**^**1**^	**Genotype**	**Acute Phase Viruses**	**Progeny PI Viruses**
180 (HQ174292)^2^	1a	6010 ^3^(JN380080)	8844 (HQ174293)
			
526 (HQHQ174294)	1b	6151 (JN380083)	8824 (HQ174295)
			
446 (HQ174297)	2	6136 (JN380082)	8827 (HQ174298)
			
446	2	6115 (JN380081)	8831 (HQ174299)
			
8833 (HQ174300)	2	8844a^4^(JN380084)	na^5^
			
Powder (JN380089)	1b	na^6^	PJ (JN380088)
			
PI99 (JN380086)	2	RNV17 (JN380090)	na^7^
			
PI103 (JN380087)	2	AM1 (JN380085)	na^7^

^1^progenitor PI virus giving rise to following acute and progeny PI viruses.

^2^strain (GenBank accession number).

^3^acute phase virus isolated from dam infected with progenitor PI virus.

^4^acute phase virus isolated from PI 8844 when cohoused with PI 8833.

^5^na: not applicable, PI calf infecting PI calf.

^6^na: not applicable, PI cow giving birth to PI calf.

^7^na: not applicable, acute infection of steer.

Similarly, the viruses AM1 and RNV17 were obtained from feedlot steers that had been exposed to PI calves PI103 and PI99, respectively (Figure1b). The viruses were isolated from serum from these steers at 13 days post-exposure [13]. Progenitor PI virus 8833 caused an acute infection in cohoused PI calf 8844. The acute phase BVDV2 virus isolated from calf 8844 was named 8844a.

### PCR and DNA sequencing

Specific regions of the genomes of the BVDV strains were PCR amplified, gel purified and DNA sequencing conducted as previously described [11]. DNA sequences were edited and assembled using Aligner software (CodonCode Corporation, USA). All numbering of nucleotides were from the ATG initiation codon of the open reading frame.

### Statistical analysis

The statistical significance of the number of nucleotide changes observed between BVDV isolates sequenced in this study was determined using Student’s T test.

## Results

### Sequencing of acute phase viruses from pregnant dams

An earlier study showed that establishment of a single persistent infection in a fetus resulted in more nucleotide substitutions then did a series of acute infections of a single virus strain that spread over a large geographic region [11]. In that study, the sequences of the progenitor PI viruses were compared to that of the progeny PI viruses, showing that there were 17 to 48 nucleotide changes in the six progeny PI viruses. To gain a greater understanding of the mechanism and timing of these changes, the ORFs of BVDV isolates from acute infections from pregnant and non-pregnant cattle exposed to PI cattle were sequenced in this study. First, comparisons were made of the progenitor and progeny PI viruses from our earlier study with the viruses isolated from the pregnant dams during the acute phase of the infection. Viruses were isolated from the dams six days following exposure to the progenitor PI calf, before infection of the fetus [9]. The relationships of these viruses are shown in Table1. In these acute isolates, the majority of nucleotide changes found in the progeny PI viruses were already present (Table2a), demonstrating that the changes were established rapidly, and before the virus crossed the placenta and infected the fetus. Additionally, each progeny PI virus contained 2 to 3 nucleotide substitutions that were unique to that virus and were not found in the progenitor PI or acute phase viruses. The number of amino acid changes in the progeny PI viruses ranged from 9 to 18 per genome that resulted in 33 to 58% of the nucleotide substitutions being nonsynonomous changes. Importantly, progeny PI virus 8824 was found to possess amino acid changes in the E2 protein that conferred antigenic change [11]. All nonsynonymous nucleotide substitutions in the E2 protein of 8824 were present in the acute phase virus. A high percentage of the nonsynonymous changes in all viruses were in the immunodominant E2 protein. A final comparison of changes introduced during pregnancy was made between the nucleotide and amino acid sequences of a virus isolated from a PI dam (type 1b virus) and the virus isolated from her PI calf shortly after birth. Here, there were 21 nucleotide substitutions that resulted in 12 amino acid differences (Table2b).

**Table 2:** Comparison of number of nucleotide and amino acid changes in BVDV isolates

**Numbers of nucleotide and amino acid changes in viruses isolated from:**								
**A. Progenitor PI, progeny PI calves and acute infections in dam**								
ProgenitorPI:Progeny PI	Nucleotide Changes (8)	Amino Acid Changes	Progenitor PI:acute	Nucleotide Changes	Amino Acid Changes	Acute:Progeny PI	Additional Nucleotide Changes^1^	Additional Amino Acid Changes
180:8844 (1a)^2^	27	9	180:6010	25	8	6010:8844	2	1
526:8824 (1b)	48	18	526:6151	45	17	6151:8824	3	1
446:8827 (2)	17	9	446:6136	15	8	6136:8827	2	1
446:8831 (2)	17	10	446:6115	14	8	6115:8831	3	2
**B. PI dam and PI calf**								
Dam:Calf								
Powder:PJ (1b)^2^	21	12						
**C. Progenitor PI calves and acutely infected cattle**								
Progenitor PI: acute								
PI99:RNV17 (2)^2^	7	2						
PI103:AM1 (2)	6	4						
8833:8844a^3^ (2)	9	7						

^1^changes unique to progeny PI virus.

^2^genotype of virus.

^3^PI calf infected by PI calf.

### Sequencing of viruses isolated from nonpregnant cattle

The two acute phase viruses isolated from steers exposed to PI calves showed a much lower number of nucleotide changes than the acute phase viruses from pregnant animals. These viruses were isolated on day 13 following exposure to the progenitor PI calf [13]. There were six and seven nucleotide substitutions in the ORFs of viruses AM1 and RNV17, respectively (Table2c). The six nucleotide changes in AM1 resulted in two amino acid changes, while two of the seven nucleotide changes in RNV17 were nonsynonomous changes. The BVDV2 virus isolated from an acute infection of a BVDV1a PI bull calf (8844) infected by a co-housed BVDV2 PI calf (8833) was isolated and found to possess nine nucleotide differences from the progenitor PI virus that resulted in seven amino acid changes (Table2c). The acutely infected calf cleared the virus and raised an antibody response against the type 2 virus. The nucleotide changes in these acute phase viruses appeared to occur randomly. When the numbers of nucleotide changes in the nonpregnant animals were compared to the number of changes observed in the viruses from the pregnant cattle, the difference was found to be statistically significant (ρ < 0.01). The genomic locations of nucleotide changes in all viruses used in this study are illustrated in Additional file 1 Table1.

It is interesting to note that there was only one nucleotide change that was found in more than one virus. Nucleotide 2647 in the E2 coding sequences of viruses 8827 and 8831 (A to G transition) resulted in an asparagine to aspartate amino acid change. These changes were found only in these progeny PI viruses and were not observed in the acute phase virus. Thus, these arose in the infection of the fetus. This particular amino acid change was observed only in these viruses and it is unclear if this is an adaptive change in crossing the placenta.

### Characterization of nucleotide changes

An analysis of the numbers and types of nucleotide substitutions found in all viruses showed that most changes were transitions and only a minority being transversions, with a ratio of 6:1 (Table3). The number of each form of transition was nearly equal with a slight bias in the number of G to A substitutions. The same is true with number of each type of transversion with a slight bias in an increased number of A:T exchanges (Table3). Further, the transition:transversion (Ts:Tv) ratios were significantly different (ρ < 0.01) when comparing viruses from pregnant and non-pregnant animals. In viruses from pregnant animals, the Ts:Tv ratio was 5.4:1 where isolates from non-pregnant animals had a ratio of 19:1. The rate of mutation of all viruses was found by the number of substitutions/1 x 10^4^ bases sequenced. In the isolates from non-pregnant animals, the mutation rate was 6 x 10^-4^ changes/nucleotide, in good agreement with published mutation rates of small RNA viruses [14]. When the same was done for isolates from pregnant animals, the mutation rate was found to be 2 x 10^-3^ changes/nucleotide, or 3.3 times higher then in the non-pregnant animal.

**Table 3:** Types of nucleotide changes observed

	**Transitions**	**Transversions**^1^						
Virus	A to G	G to A	C to T	T to C	A:C	A:T	G:C	G:T
6151	10	12	6	5	0	2	2	5
6136	1	6	7	2	0	2	0	0
6115	3	5	4	1	1	1	0	0
6010	8	7	4	4	1	2	0	0
PJ^2^	3	6	3	5	1	0	2	0
Total^3^	25	36	25	17	3	7	4	5
								
AM1	1	1	3	1	0	0	0	0
RNV17	3	1	0	3	0	0	0	0
8844a	1	2	0	4	1	0	0	0
Total	4	4	3	8	1	0	0	0
								
Total^4^	30	40	27	25	4	7	4	5

^1^represents both pyrimidine:purine and purine:pyrimidine.

^2^Virus PJ was included here because its source was a pregnant animal.

^3^Transition:transversion ratio of 5.4:1.

^4^Transition:transversion ratio of 19:1.

**Figure 2:**
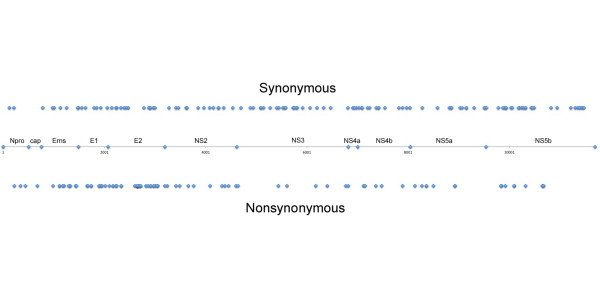
**Location of all nucleotide substitutions identified in all BVDV isolates sequenced.** The synonymous nucleotide changes are illustrated on the top and the nonsynonymous changes illustrated on the bottom of the figure. The line in the center represents the BVDV genome and the location of the processing points that result in formation of the mature viral proteins. The names of the individual mature proteins are shown.

The positions within the BVDV genome where both synonymous and non-synonymous nucleotide changes were found were plotted and are illustrated in Figure2. Plotting of synonymous changes revealed that these changes were essentially randomly distributed throughout the genome with only small regions having no changes. The positions of nonsynonymous nucleotide changes, however, showed a bias toward changes in the structural proteins, especially in the E^rns^ and the E2 proteins, probably in locations where amino acid changes were more readily tolerated. There were fewer changes observed in the nonstructural proteins. The NS2 encoding region contained the largest number of nonsynonymous nucleotide changes of the nonstructural proteins.

## Discussion

It is well established that BVDV isolates show considerable variation in nucleotide sequence, even within subgenotypes. However, it has not been determined how and when this genetic variation arises. It was proposed that variability in nucleotide sequence arises primarily during acute infections with immune pressure being the major driving force behind changes in the immunogenic structural proteins [15]. Changes in the nonstructural proteins come about more slowly and are due primarily to the error-prone RNA-dependent RNA polymerase.

Previous studies of genetic change in BVDV using sequencing of portions of the 5’ UTR and E2 protein of the BVDV genomic RNA revealed that nucleotide substitutions were introduced slowly [5,16]. The choice of the E2 protein in these earlier studies, based on data presented here, was an appropriate choice for analysis of genetic changes because of the greater number of changes. However, the small size of the genomic region analyzed provided only limited analysis. In our earlier study [11], we found that sequencing of the entire ORF provided a more complete overview. Furthermore, this showed that more nucleotide substitutions were found in the BVDV genomic RNA during a single ‘in vivo passage’ in the establishment of a persistent infection in a calf in utero than in a series of acute infections caused by a single strain of BVDV over a large geographic region and greater than a year’s time. These data indicated that infection of pregnant animals may be the greater source of genetic variation. Unfortunately, this analysis did not answer the question of when the changes were introduced in pregnant cattle; specifically, whether they occurred during the acute infection of the dam or in crossing the placenta and infection of the fetus. To address this, the ORF of viruses isolated from the acute infections of the dams from this previous study were sequenced and compared to the sequences of the progenitor and progeny PI viruses to determine when and to what extent that variation in BVDV genomic sequences occurred. This clearly demonstrated that the majority of the genetic changes were introduced during the acute infection of the dam. Within six days of exposure to the progenitor PI calf, the virus isolated from serum possessed the majority of the changes identified in the progeny PI virus. The remaining two to three nucleotide changes found in the progeny PI viruses were introduced during the infection of the fetus. Importantly, the acute phase virus was altered before the infection of the fetus. Also, based on the number of changes in the genome of the progeny PI from that of the acute phase virus from the dam, it appears that the fetus can be considered a non-pregnant host, thus exhibiting low numbers of additional nucleotide substitutions. One particularly surprising finding in this study was the number of nucleotide changes that were introduced in the progeny PI virus infecting the calf of the PI dam. It was expected that the virus of the dam would infect the fetus with little resulting genetic change because of lack of adaptive immunity in the dam or fetus, and the large amount of virus that is continuously present. It is not clear at this point why this occurred.

The data presented here revealed that nucleotide substitutions occurred nearly randomly throughout the genomic RNA of BVDV with nonsynonymous changes being more limited in the regions of the genome where they may occur (Figure2). The occurrence of nonsynonymous changes was biased to specific genomic regions, primarily the structural proteins, where amino acid changes are more easily tolerated. There appeared to be specific locations where change was not tolerated, most likely to conserve critical functions of the proteins. Examination of the types of nucleotide substitutions showed that there is not a great difference in the numbers of the four different types of transitions (Table3). Transitions outnumbered transversions by more than 6:1, implicating misincorporation of nucleotides by the RNA-dependent RNA polymerase [17,18]. When the total numbers of nucleotide substitutions were compared between acute viruses isolated from pregnant animals and acute viruses from non-pregnant animals, there was a 3.3 fold increase in nucleotide substitutions in pregnant animals over non-pregnant animals with a range of 2 to 6 fold. In the non-pregnant animals, the BVDV isolates had a mutation rate of 6 x 10^-4^ substitutions per nucleotide, while substitution rates in the pregnancy-associated viruses were 1.9 x 10^-3^ substitutions per nucleotide, or an average of 3.3 fold higher. This clearly shows that there was some mechanism present in pregnant animals that brought about or allowed greater genetic variability to be introduced into the BVDV genome.

There may be two possible reasons for the observation of a greater number of nucleotide changes in the genomic RNAs of BVDV in pregnant animals. First, there may be a protective mechanism that is more active in pregnant cattle that introduces mutations into the BVDV RNA genome in an attempt to stop or limit the infection. This type of mechanism that may resemble the APOBEC cytosine deaminases, may enzymatically alter nucleotides to introduce lethal genetic changes in a manner similar to those introduced into the genomes of lentiviruses and hepatitis B virus [19-24]. Analysis of the form of nucleotide changes found in BVDV strains (Table3) indicated that this type of enzymatic mutagenesis was unlikely to be the source of genetic change in these viruses. There was not a preponderance of changes that would result from cytosine to uridine changes. In fact, most changes were transitions that resembled the misincorporation of nucleotides by the RNA-dependent RNA polymerase where guanosine may bond with uridine, similarly to that reported in poliovirus, foot and mouth disease virus, and cucumber mosaic virus [17,18,25].

The second and the more probable mechanism lies in the BVDV quasispecies of the infecting progenitor PI calf and the genetic bottleneck created by crossing protective barriers of the naïve animal [26] which may vary based on reproductive status. Multiple physical layers must be breached in order to establish a productive infection. In the case of BVDV, the mucous secretions covering the nasal mucosa must be penetrated, followed by infection of the underlying cells. The innate immune mechanisms of these cells must be overcome, followed by penetration of the virus into the underlying cell layers. Finally, spread into the vascular system must be achieved for widespread dissemination. These physical and immunological barriers take a considerable toll on the infecting virus where only a small number survive, acting to reduce quasispecies genetic diversity in a stochastic manner. With each virus in the quasispecies differing from the population consensus sequence by only a few nucleotides, infection by a small number of viruses would, in all likelihood, result in a new, slightly different population consensus. Two models were proposed by Pfeiffer and Kirkegaard [27] that may explain genetic change in BVDV. The “tough-transit” model proposes that viruses have a difficult time passing the natural barriers and innate immunity of the host and the few viruses that succeed in bypassing the barriers establish the new viral population. The “burned-bridges” model proposes that viruses have little difficulty bypassing the protective barriers and the first to do so quickly establish an antiviral state that inhibits further infection by following viruses. The data presented here suggests that the “tough-transit” model may be more applicable. BVDV normally have difficulty passing the natural barriers, as evidenced by the few changes observed in viruses from non-pregnant animals. The few surviving viruses would quickly amplify and, depending on the sequence of these viruses, establish a new population consensus sequence. In pregnant animals, where immunity is already altered because of the pregnancy, the innate immune system may be somewhat leaky or less effective at stopping infection, allowing a greater number of infecting virus particles to establish a productive infection. This results in a population consensus with a larger number of nucleotide differences from the population mean of the infecting animal, all being dependent on the number of infecting viruses and which and how many became prevalent in the new quasispecies. This view was supported by the findings that the acute virus isolated from a pregnant dam already possessed a large number of nucleotide changes from the progenitor PI virus while the progeny PI virus showed only 2 or 3 nucleotide changes from the acute phase virus, resulting from infection of a non-pregnant animal (fetus) with intact innate immunity. Also, the viruses from the acutely infected steers (Figure1b) possessed few additional changes.

This study provided results that defined where and when nucleotide changes are generated or introduced into BVDV genomic RNAs. However, to understand the mechanism of genetic change in greater detail, a deeper analysis of the diversity in the viral quasispecies present in each virus population is needed. Future experiments will require deep sequencing of each quasispecies to look for the presence and prevalence of the specific mutations found in the new quasispecies to provide a more detailed understanding of genetic change in BVDV.

## Competing interests

The authors declare that they have no competing interests.

## Author’s contributions

JDN and MDG conceived the study and participated in its design. MDG, BWN and SDM conceived and conducted the study where progenitor, progeny and acute phase viruses were isolated. JDN and JFR conducted sequence analysis of BVDV isolates and assembled data. All participated in writing of the manuscript. All authors read and approved the final manuscript.

## Disclaimer

Mention of trade names or commercial products in this article is solely for the purpose of providing specific information and does not imply recommendation or endorsement by the U.S. Department of Agriculture.

## Supplementary Material

Additional file 1 Table 1. Positions of nucleotide changes in BVDV isolates. Click here for file
