# Supplementary material for: Greater numbers of nucleotide substitutions are introduced into the genomic RNA of bovine viral diarrhea virus during acute infections of pregnant cattle than of non-pregnant cattle
Source: Virol J. 2012 Aug 6;9:150. doi: 10.1186/1743-422X-9-150 (PMC3487799; doi:10.1186/1743-422X-9-150)
Supplement: Additional file 1 — Table 1. Positions of nucleotide changes in BVDV isolates. [file 1743-422X-9-150-S1.docx]

| **Supplementary Table 1. Positions of nucleotide changes in BVDV isolates** | | | | | | |
| --- | --- | --- | --- | --- | --- | --- |
|  | |  | |  | |  |
| **A. Progenitor PI, Progeny PI calves and acute infections in dams** | | | | | | |
| **Progenitor:Acute** | **Nucleotide Positions** | | **Acute:Progeny** | | **Nucleotide Positions** | |
| 180:6010 | 210, **1180, 1741**, 1843, | | 6010:8844 | | **1898**, 7197 | |
|  | 2055, 2151, 2244, 2394, | |  | |  | |
|  | **2863**, **3061**, 3466, 3963, | |  | |  | |
|  | 5274, 5748, 6066, **6178**, | |  | |  | |
|  | **6403**, 7416, 8040, 9150, | |  | |  | |
|  | **9830**, 10062, **10093**, | |  | |  | |
|  | 10182, 11382 | |  | |  | |
| 526:6151 | 783, **988**, 1008, 1129, | | 6151:8824 | | 3003, 3853, **9925** | |
|  | **1203**, **1315,** **1330,** 1624, | |  | |  | |
|  | **1660,** **2680, 2714, 2912**, | |  | |  | |
|  | **2917,** 2970, **2978,** **3194,** | |  | |  | |
|  | **3487, 4262** **4456,** **4645,** 4701 | |  | |  | |
|  | 4881, 5088, 5148, 5562, | |  | |  | |
|  | 5583, 5733, 5811, **6820,** | |  | |  | |
|  | 6978, 7245, 7422,7818, | |  | |  | |
|  | 9072, **9851,** 9876, 10245, | |  | |  | |
|  | 10332, **10681,** 10827, | |  | |  | |
|  | 10947, 11301, 11352 | |  | |  | |
| 446:6136 | **1118, 1952, 2605, 2825,** | | 6136:8827 | | **2647***, 11211 | |
|  | **3008**, 3279, 4152, 5406, | |  | |  | |
|  | 5527, 6813, 7089, **8365**, | |  | |  | |
|  | 8583, **8932, 10651** | |  | |  | |
| 446:6115 | **220**, 1251, **2330**, 2428, | | 6115:8831 | | **2171**, **2647***, 7971 | |
|  | 2904, **3245**, **4087, 4381**, | |  | |  | |
|  | **5608**, 6204, **6772**, 7032, | |  | |  | |
|  | **7477**, 11448 | |  | |  | |
|  |  | |  | |  | |
| **B. PI dam and PI calf** | | |  | |  | |
| Powder:PJ | | 126, 975, **1430, 14312,** |  | |  | |
|  | | **1904, 1933**, 2469, **2653**, |  | |  | |
|  | | **2714**, 2778, **4325, 4597**, |  | |  | |
|  | | **8270, 8360, 8495**, 8820, |  | |  | |
|  | | **8921**, 9027, 9153, 9780, |  | |  | |
|  | | 9909 |  | |  | |
|  | |  |  | |  | |
| **C. Progenitor PI calves and acutely infected cattle** | | | | |  | |
| PI99:RNV17 | | **1292,** 1473, 1929, **2648,** | |  |  | |
|  | | 7893, 9549 | |  |  | |
| PI103:AM1 | | **1115, 3038**, 4356, 7098, | |  |  | |
|  | | **8377,** **10666** | |  |  | |
| 8833:8844a | | 2171, 2229, 3764, 7894, | |  |  | |
|  | | 8352, 8856, 9942, 10317, | |  |  | |
|  | | 10324 | |  |  | |
| ^1^nucleotide positions in bold font represent nonsynonomous changes | | | | | | |
| ^2^nucleotides 1430 and 1431 in same codon, resulting in 1 amino acid change | | | | | | |
